# Comparison of the Clinical Outcomes of Guillain Barre Syndrome Based on Electrophysiological Subtypes in Pakistani Children

**DOI:** 10.7759/cureus.8052

**Published:** 2020-05-11

**Authors:** Asmat Parveen, Sabeen Abid Khan, Sidra Talat, Syeda Namayah Fatima Hussain

**Affiliations:** 1 Pediatrics, Benazir Bhutto Hospital, Rawalpindi, PAK; 2 Pediatrics, Shifa College of Medicine, Shifa Tameer-E-Millat University, Islamabad, PAK; 3 Pediatrics, Holy Family Hospital, Rawalpindi, PAK; 4 Medicine, Liaquat National Hospital and Medical College, Karachi, PAK

**Keywords:** guillain barre syndrome, acute flaccid paralysis, children

## Abstract

Objective

Guillain Barre syndrome (GBS) is an autoimmune-mediated, acute, symmetrical, flaccid paralysis. Guillain Barre syndrome has different electrophysiological types that carry prognostic significance and tend to differ between adults and children. This study aims to compare the clinical outcome of Guillain Barre syndrome in Pakistani children based on their electrophysiological types to help in understanding and predicting the prognosis.

Study design

Observational comparative study

Place & duration

The pediatric department, Shifa International Hospital, Islamabad; all patients with Guillain Barre syndrome seen between 2012 and 2019

Method

All children aged one to 16 years in whom Guillain Barre syndrome was diagnosed based on clinical history, examination, and electrophysiological findings. Institutional review board (IRB) approval was taken and data entered on the designed questionnaire. Chi-square and non-parametric tests were applied for significant association.

Results

Twenty-three children were included in the study. Of these, 14 were males (60.9%) while the mean age was 5.8 (+4.5) years. Acute inflammatory demyelinating polyneuropathy (AIDP) was found to be the predominant type (9; 39.1%) followed by acute motor and sensory axonal neuropathy (AMSAN) (6; 26.1%), Acute motor axonal neuropathy (AMAN) was diagnosed in four (17.4%) patients. Six (26.1%) patients needed mechanical ventilation and 10 patients (43.5%) required intensive care unit (ICU) care. The majority of the patients (18; 78.3%) received intravenous immunoglobulin (IVIG).

Conclusion

The study highlights varied electrophysiological types of GBS in Pakistani children, which differ in predominance from previous studies. However, various indicators of poor outcomes that are highlighted in adults, including the older age group, need for mechanical ventilation, and electrophysiological evidence of axonal degeneration, were not significant predictors of outcome in children.

## Introduction

Guillain Barre syndrome (GBS) is an acute, symmetrical, immune-mediated, ascending paralysis of multifactorial etiology mostly caused by a preceding infection [1-2]. GBS is characterized by progressive motor weakness of limbs, areflexia, and albuminocytological dissociation in cerebrospinal fluid (CSF) [3]. The symptoms progress over two weeks and reach the maximum neurological deficit at around four weeks [2]. The overall incidence of Guillain Barre syndrome is 1.1 to 1.8/100,000/year; however, there is insufficient data available regarding its incidence in Pakistan [1]. Electrodiagnostic studies play a significant role in the early detection and classification of Guillain Barre syndrome in the first week after the symptoms appear and, therefore, play an important role in treatment, as timely intervention reduces morbidity and mortality [1,4]. They also help in predicting functional outcomes and management [2,4-5]. Four main types of GBS are acute inflammatory demyelinating polyneuropathy (AIDP), acute motor axonal neuropathy (AMAN), acute motor and sensory axonal neuropathy (AMSAN), Miller Fisher syndrome (MFS), and mixed variety [2,5].

Previous studies from western countries show that AIDP is the most common subtype of GBS followed by AMAN, which is reported to have a severe presentation and worse outcome as compared to the demyelinating type [4,6-8]. In Pakistani literature, axonal is the most common type reported in adults from Punjab while it is the demyelinating type from Sindh [1,9]. However, our studies do not show a difference in functional outcomes in the axonal and demyelinating subtypes in contrast to the western population. Moreover, the duration of hospital stay and the need for mechanical ventilation also show a similar pattern in the axonal and demyelinating types in adults and negative stool cultures for Clostridium jejuni are some of the atypical features seen in our population [10]. Intravenous immunoglobulins (IVIGs) and supportive care are the mainstays of treatment whereby IVIG and plasma exchange have equal efficacy and steroids have no role as a single therapy [11]. Limited data are available regarding frequency, clinical course, and outcomes of patients based on the different electrophysiological subtypes of Guillain Barre syndrome in the pediatric age group in Pakistan to help us predict functional outcomes and guide management. Therefore, the current study aims to compare the clinical presentations and outcomes of patients based on the electrophysiological subtypes of Guillain Barre syndrome in children.

## Materials and methods

All patients admitted in the pediatric department of Shifa International Hospital between 2012 and 2019 with a diagnosis of Guillain Barre syndrome were evaluated. Patients of both genders and aged from one to 16 years were included in the study. The available data were checked for history, clinical findings, history of preceding infection, recent vaccination, the severity of illness, relevant investigations, electrophysiological subtypes, management and residual disability at the time of discharge, and follow-up at three months. Information was filled on a self-designed questionnaire. Institutional review board approval was taken.

The data were entered and analyzed using SPSS version 23 (IBM Corp, Armonk, New York). For quantitative variables, such as age, height, weight, length of hospital stays, and duration of illness, means and standard deviation were calculated. Frequencies and percentages will be measured for qualitative variables such as gender. Chi-square test and non-parametric tests like Kruskal Wallis were used for any significance. The level of significance will be considered (p-value ≤0.05). Moreover, for effect modifiers, a comparison was done by stratifying the sex and age of the child.

## Results

A total of 23 patients with GBS were included in this study. Among these were 14 (60.9%) males and nine females (39.1%) while the mean age was 5.8 (+4.5) years. Sixteen (69.6%) children belonged to urban areas and seven (30.4%) were from rural areas. The mean duration of illness was 6.3 (+5.9) days, varying from one to 30 days. Hospital stay ranged from two to 32 days with a mean (SD) of 6.7 days (6.0). There was a history of antecedent infection in 18 patients (78.3%) with upper respiratory tract infection being the most common cause (11/18, 61.1%) followed by gastrointestinal infection (7/18, 38.9%) while one patient had both. Out of 23 patients, one (4.3%) had a history of recent vaccination (p 0.21).

All patients reported symmetrical ascending limb weakness (100%). No progression of weakness was seen in 17 (73.9%) while five (21.7%) had slow progression and one (4.5%) was documented to have rapid progression. There was decreased tone in the upper and lower limbs in 21 (91.3%) patients. Areflexia in all limbs was seen in 14 (60.9%) children while nine (39.1%) were noted to have absent reflexes in the lower limb only. One (4.5%) patient had a low Glasgow Coma Scale (GCS).

Electromyography and nerve conduction studies were undertaken by all patients. AIDP was found to be the predominant type (39.1%) of GBS. The other subtypes are listed in Table 1. The mean cerebrospinal fluid (CSF) protein and white blood cell (WBC) count were 46.7 mg/dl and 6.3 x 109/L, respectively. Nine out of 23 (39.1%) patients were found to have albuminocytological dissociation in the CSF. Six (26.1%) patients needed mechanical ventilation and 10 patients (43.5%) required ICU care. Stool analysis of 14 (60.9%) patients was done; all were negative for Campylobacter jejuni. The majority of the patients (18; 78.3%) received IVIG while none was given plasmapheresis. Residual disability at discharge and follow-up is given in Table 2. However, one patient was not included in this since he was still under treatment at the hospital.

**Table 1:** Classification of different subtypes of GBS GBS: Guillain Barre syndrome; AMAN: acute motor axonal neuropathy; AIDP: acute inflammatory demyelinating polyneuropathy; CIDP: chronic inflammatory demyelinating polyneuropathy; AMSAN: acute motor and sensory axonal neuropathy

GBS Types	Frequency	Percentage
AMAN	4	17.4
AIDP	9	39.1
CIDP	1	4.3
AMSAN	6	26.1
Miller Fischer Variant	1	4.3
Unclassified	2	8.7
Total	23	100.0

**Table 2:** Comparison of the major subtypes of GBS The table shows a comparison of the three major subtypes of GBS only. Less common varieties are not shown here, which were found in three male patients: one male patient had CIDP, Miller Fischer variant, and unclassified each, therefore, making a total of 14 male patients. GBS: Guillain Barre syndrome; CIDP: chronic inflammatory demyelinating polyneuropathy

Variables	AIDP	AMSAN	AMAN	p-value
Age (year-old)	2.00 + 0.00	4.00 + 0.00	1.00 + 0.00	0.23
Male	5 (55.6%)	3 (50.0%)	3 (75.0%)	0.85
Antecedent Infection	8 (88.9%)	4 (66.7%)	3 (75.0%)	0.77
URTI	4 (44.4%)	3 (50.0%)	1 (25.0%)	0.85
Diarrhea	4 (44.4%)	2 (33.3%)	2 (50.0%)	1.00
Cranial Nerve Involvement	0 (0.0%)	0 (0.0%)	1 (25.0%)	0.21
Autonomic Dysfunction	2 (22.2%)	0 (0.0%)	0 (0.0%)	0.68
Bulbar Muscle Weakness	3 (33.3%)	3 (50.0%)	2 (50.0%)	0.85
Pain & Paresthesia	4 (44.4%)	1 (16.7%)	1 (25.0%)	0.69
Respiratory insufficiency	2 (22.2%)	2 (33.3%)	2 (50.0%)	0.81
ICU Care	5 (55.6%)	3 (50.0%)	2 (50.0%)	1.00
Residual Disability at Discharge	7 (77.8%)	6 (100.0%)	3 (75.0%)	0.68
Residual Disability at Follow-Up	2 (10.5%)	0 (0.0%)	0 (0.0%)	0.09

## Discussion

GBS is rare but the commonest cause of acute flaccid paralysis worldwide and has a variable prognosis. However, in Pakistan, it is difficult to ascertain this because of the high number of poliomyelitis cases confirmed last year by the World Health Organization (WHO) Polio Surveillance team. Our study reports 23 patients of GBS during the study period of seven years. All our patients underwent polio screening and were confirmed negative.

Our results show a male gender predominance at a ratio of 1.5:1. This is consistent with studies done in both children and adults worldwide. This finding of male gender predilection remains unexplained in the literature. The mean age of our patients was 5.8 (+4.5) years. Another study from Pakistan has given a mean age of 6.7 years. GBS has been reported more often in patients older than 18 years and associated with severe disability and poor prognosis [12]. Our study shows AIDP as the most common electrophysiological subtype of GBS (39.1%). Shafqat et al. also reported the demyelinating variety as the most common among his adult patients [10]. The study by Chand et al. in Karachi also revealed the demyelinating variety to be the most common (58%) in children [3]. Similarly, Alvi et al. had 33% of children with the demyelinating variety while 44% had demyelinating with axonal involvement [13]. A recent study at the Children’s Hospital Lahore also reported AIDP as the most common variant (67%) [7]. AIDP is common in the western population while AMAN has a higher incidence in East Asia (China and Japan). We also report one case of CIDP with an acute presentation. Acute GBS-like presentation of CIDP is rare but reported in the literature [14]. It is associated with a chronic relapsing course and residual disability, as seen in our patient.

The second most common subtype was AMSAN, diagnosed in six (26.1%) patients. This contrasts with other studies from Pakistan that report AMAN as the second most common type. Half (50%) of the patients with this type had bulbar muscle weakness and needed ICU care and all had a residual disability at discharge. AMSAN is generally associated with gradual and incomplete recovery [15].

AMAN was diagnosed in four (17.4%) patients. One of these patients had a history of vaccination when he received the oral polio vaccine (OPV). The incidence of AMAN varies geographically from less than 10% in the west to over 40% in East Asia and is more commonly reported in children. AMAN is associated with a rapidly progressive disease but a good recovery [16]. In our study subjects, 75% had a residual disability at discharge, one patient (4.3%) had the Miller Fisher variant while two (8.7%) had an unclassified disease. MFS is reportedly rare in children [17].

The most common preceding infection was reported to be an upper respiratory infection followed by diarrhea. In the literature, GBS is followed by a gastrointestinal or an upper respiratory infection in 70% of the cases [1]. Campylobacter jejuni, the most isolated organism, is associated with the axonal type, severe disability, and a worse prognosis [1-2]. None of our patients had a stool culture positive for campylobacter jejuni. Similar findings were reported by a study done in Karachi [8].

All patients had symmetric ascending paralysis. Bulbar weakness was reported in nine patients (39.1%). Pain and paresthesia were reported by nine (39.1%) patients while six (26.1%) had respiratory insufficiency and needed mechanical ventilation. Cranial nerve involvement is less frequently seen in children, consistent with our results. Various indicators of poor outcome implicated by several studies in adults include the older age group, need for mechanical ventilation in the acute phase, preceding diarrhea, and electrophysiological evidence of axonal degeneration. However, no significant predictors of outcome were identified in children; this is consistent with the result seen in Pakistani adults.

There was no mortality in our study population. However, the mortality rates range from 5%-10% and are a significant cause of mortality and morbidity in adults [18]. Four (18.2%) patients completely recovered at discharge while 16 (81.4%) had a residual disability. Out of these eight patients lost to follow-up, seven (43.7%) patients had improved while three (18.7%) patients still had a residual disability. GBS in children carries a good prognosis where 80% and 84% of patients walk independently at six months and one year, respectively.

## Conclusions

The study highlights that GBS is a rare but important differential in acute flaccid paralysis, especially in the Pakistani context, with emerging cases of poliomyelitis. The electrophysiological subtypes of GBS in Pakistani children also differ in predominance from previous studies. AIDP was the most common type, followed by AMSAN and AMAN. There was no mortality reported in our study population. However, various indicators of poor outcomes highlighted in adults, including older age group, need for mechanical ventilation, and electrophysiological evidence of axonal degeneration, were not significant predictors of outcome in children.
